# Assessing vitamin D as a biomarker in inflammatory bowel disease

**DOI:** 10.1002/jgh3.13010

**Published:** 2023-11-27

**Authors:** Fiona Yeaman, Anke Nguyen, Joshua Abasszade, Sanjana Gupta, Sally Bell, Gregory Moore

**Affiliations:** ^1^ Department of Gastroenterology and Hepatology Monash Health Clayton Victoria Australia; ^2^ Department of Medicine, School of Clinical Sciences Monash University Clayton Victoria Australia

**Keywords:** biomarker, fecal calprotectin, inflammatory bowel disease, vitamin D

## Abstract

**Background and Aim:**

A reliable serum biomarker for inflammatory bowel disease (IBD) activity is needed. Vitamin D is involved in inflammation and has been demonstrated to be low in IBD patients with active disease. It is routinely measured in IBD patients. Therefore, vitamin D may have a role as a serum biomarker in IBD. This study aims to investigate whether serum vitamin D may be useful as a biomarker in IBD in a real‐world IBD population.

**Methods:**

Patients were identified by review of fecal calprotectin (FCP) results, and those who had a clinical review, vitamin D test, and FCP performed within 3 months were included. Clinical scores were calculated from chart review. Nonparametric tests were used to investigate vitamin D and FCP levels, serum biomarkers, and clinical scores.

**Results:**

Of 616 patients identified, 325 episodes of matched vitamin D level and biomarker data were obtained. A statistically significant correlation was found between vitamin D levels and FCP levels for all patients (*r* = −0.19 [*s* –0.29 to −0.080], *P* < 0.001]. This remained true when patients were divided into IBD subsets. Low vitamin D was associated with partial Mayo scores and C‐reactive protein (CRP) to albumin ratio in ulcerative colitis, and CRP and CRP/albumin ratio in Crohn's disease.

**Conclusion:**

Vitamin D level is negatively correlated with FCP and it may be considered as an adjunct biomarker at this stage. A prospective study would be beneficial to investigate further correlations between vitamin D and existing biomarkers of inflammation in IBD.

## Graphical abstract

Vitamin D is known to be involved in inflammation and low in active inflammatory bowel disease (IBD). A specific, sensitive and low‐cost serum biomarker for activity in IBD does not currently exist. This study investigates this potential association and found that vitamin D levels negatively correlated with fecal calprotectin levels and variably correlated with known serum biomarkers with some significant correlations seen, particularly in the ulcerative colitis cohort.

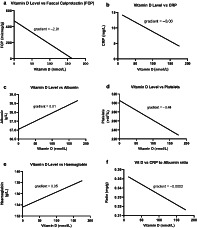

## Introduction

Optimal management of inflammatory bowel disease (IBD) is a key area of interest, and treatment targets have been under the microscope.^1^ Although gastrointestinal (GI) symptoms are common in active IBD, they are not always present and, when present, they are not always due to inflammation. The gold standard for disease activity detection is endoscopy, but this is invasive, expensive, and has relatively low patient acceptance unless symptoms are severe. A specific, sensitive, and reliable serum biomarker for IBD activity does not exist currently. C‐Reactive protein (CRP) is used, but a portion of patients have either a modest or absent CRP response, particularly those with ulcerative colitis (UC).^1^, ^2^ The composite marker CRP/albumin ratio has also been shown to be useful in predicting disease outcomes.^3^ Fecal calprotectin (FCP) test is noninvasive and has a sensitivity of 0.88 (95% CI: 0.84–0.90) for active IBD^4^ but is not always funded and requires stool collection, thus lowering patient acceptability.^5^ Cross‐sectional imaging involves radiation exposure and is primarily useful for transmural complications of Crohn's disease (CD).^6^ Intestinal ultrasound (IUS) can detect active disease but is not routinely available in many centers.^7^

Vitamin D has been shown to play a role in inflammation in multiple conditions including sepsis, chronic kidney disease, and IBD.^8^, ^9^, ^10^ The mechanism behind this is believed to be due to regulation of the production of inflammatory cytokines and inhibition of the proliferation of proinflammatory cells.^10^ Low vitamin D has been shown to be associated with active IBD in retrospective studies both in Australia and internationally, suggesting its potential role as a biomarker.^11^, ^12^, ^13^, ^14^, ^15^ Low vitamin D levels correlate with worse outcomes and more complex disease in IBD.^16^, ^17^ Vitamin D has also been proposed as a treatment for IBD, but studies to date have shown it to be beneficial only as an adjunct.^16^, ^18^, ^19^ Vitamin D testing is inexpensive (approximately $8 AUD per test compared to $50–80 for calprotectin) and results are consistent.^20^

There is a paucity of data assessing whether serum vitamin D is useful as a biomarker in the clinical setting in IBD. This study aims to investigate whether serum vitamin D may be useful as a biomarker by establishing how it correlates with FCP, other biomarkers, and clinical scores.

## Design and methods

Quality assurance ethical approval was obtained from the Monash Health Human Research Ethics committee (RES‐19‐0000‐957Q). Patient consent was not required given routine patient data were collected retrospectively and de‐identified as part of an audit process. Retrospective data was collected via a chart review of the hospital pathology system and medical records. Patients were initially selected by using the pathology database of those who had their FCP measured in 2019. From this initial cohort, IBD patients with a clinical assessment, FCP, and blood tests including vitamin D level within 3 months were identified from 2015 to June 2020. Vitamin D status is routinely monitored in blood samples in this clinic, given the community prevalence of vitamin D deficiency and noted negative correlations with active inflammation. Given the retrospective nature of this study, we could not take into account the seasonal variation, and matched patient data did not consistently represent different times of year.

Demographic data, IBD subtype, treatment, and symptoms were obtained from the clinical records of patients. FCP is funded by the hospital and is routinely used in monitoring IBD patients at this tertiary clinic. Test results for calprotectin, inflammatory markers, and 25‐hydroxy‐vitamin D (a marker of vitamin D status) were sourced from the pathology laboratory information system. Vitamin D levels were assessed using competitive immunoassay. Endoscopic assessment was not relevant to this noninvasive, clinic‐based study.

Data were subsequently de‐identified, tested for normality, and analyzed using GraphPad Prism v. 9. Demographic data were analyzed using descriptive statistics and frequency distributions. The vitamin D levels were compared using Spearman rank correlation with FCP, CRP, albumin, hemoglobin, and platelets. CRP/albumin ratio and Harvey Bradshaw Index (HBI) for CD patients and partial Mayo score (pMayo) for UC patients were also calculated and compared with vitamin D status. Receiver operating characteristic (ROC) curves were used to determine what vitamin D level was sensitive and specific for an elevated FCP. Intra‐patient variability was also investigated. The data did not meet tests of normality, and therefore change between patients evaluated with Spearman rank correlation and Wilcoxon matched pairs rank test was used to compare individual patient results at two different time points. Elevated FCP determined to be clinically relevant was set at a value >150 μg/g.^21^

## Results

The initial cohort comprised 616 patients. Three‐hundred and twenty‐five patient episodes with matched FCP and blood tests including vitamin D, CRP, albumin, hemoglobin, and platelets were identified. This consisted of 81 CD patients with 184 care episodes (52%) of matched data and 67 UC patients with 127 care episodes (43%) of matched data. There were 14 IBD‐unspecified care episodes with matched data (7 patients, 4.5%). Baseline characteristics for patients characterized by IBD diagnosis are listed in Table 1. Further breakdown of disease by location is given in Table 2.

**Table 1:** Baseline characteristics

	All patients (*n* = 155)	CD (*n* = 81)	UC (*n* = 67)	IBD‐U (*n* = 7)
Sex (% male)	45	46	43	71
Age (median, years) [IQR]	38 [25–52]	34 [23–50]	38 [28–54]	57 [50–60]
Disease duration (median, years) [IQR]	5.9 [2.3–12]	7.8 [2.6–15]	4.6 [1.2–11]	4.9 [2.8–7.5]
Biologic therapy (%)	46	52	50	N/A^†^
Vitamin D supplementation (%)	25	13	13	N/A^†^

^†^ Data available only for one patient with IBD‐U.

CD, Crohn's disease; IBD‐U, inflammatory bowel disease unclassified; IQR, interquartile range; UC, ulcerative colitis.

**Table 2:** IBD phenotype breakdown

**Ulcerative colitis disease location, *n* (%)**			
Proctitis	9 (13)		
Left‐sided	27 (40)		
Pancolitis	31 (46)		
**Crohn's disease location, *n* (%)**			
Ileal	27 (33)	Perianal	21 (26)
Colonic	15 (19)	Upper GI	Not collected
Ileocolonic	39 (48)		
**IBD‐U, *n* (%)**			
Colonic	7 (4.5)		

IBD‐U, inflammatory bowel disease unclassified.

Over half of the patient episodes were recorded while on biologic therapy: 52% in CD and 50% in UC. Treatment data was not available for 9% of patients. Smoking data was not recorded for 47% of patients, but of those with records, the majority were CD patients (19%). Also, 4% of UC patients were recorded as smokers during their clinic reviews, and 13% of both CD and of UC patients were recorded as taking vitamin D supplementation. Disease activity over time fluctuated, and active disease was noted on clinical review criteria (defined as partial Mayo >2 in UC and HBI > 5 in CD) in 51% of UC patients and 22% of CD patients.

Median vitamin D level for all patients across all episodes of care was 56 nmol/L (interquartile range [IQR] 42–72) (reference range 50–250 nmol/L). There was no difference between smokers (56 nmol/L, IQR 39–66) and non‐smokers (55 nmol/L, IQR 41–66; *P* = 0.98), as well between biologic therapy (57 nmol/L; IQR 47–72) no biologic therapy (55 nmol/L; IQR 41–71; *P* = 0.19).

**Figure 1:**
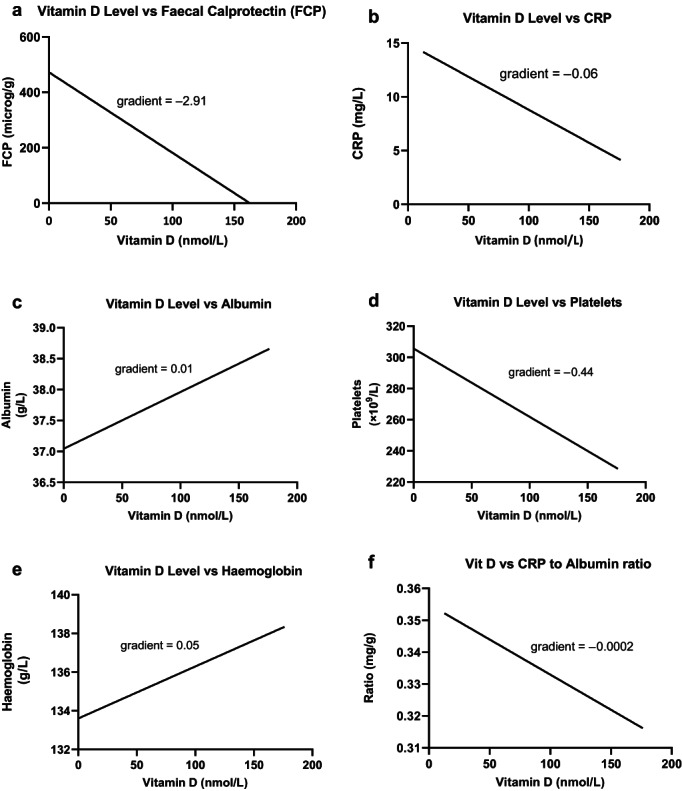
Linear regression of vitamin D *versus* (a) FCP, (b) CRP, (c) albumin, (d) platelets, (e) hemoglobin, and (f) CRP/albumin ratio. CRP, C‐reactive protein; FCP, fecal calprotectin.

Linear regression plots between vitamin D and the other biomarkers are shown in Figure 1. This showed a statistically significant negative correlation when comparing vitamin D level and FCP, platelet count, and CRP but not albumin or hemoglobin. The corresponding statistical analyses are shown in Table 3. When all patient episodes were assessed, a statistically significant negative correlation was found between vitamin D levels and FCP levels (*r* = −0.19 [−0.29 to −0.080], *P* < 0.001). This remained true when patients were divided into CD and UC subsets. Vitamin D levels were found to negatively correlate with CRP and platelet counts when all patients were considered together, but when split into CD and UC, this did not remain statistically significant. There was no correlation between vitamin D levels and albumin or hemoglobin for all patients or the CD subset. UC patients had a statistically significant positive correlation between albumin level and vitamin D level. Vitamin D was also significantly correlated with CRP/albumin ratio for all patients and for UC patients, but not for CD patients alone.

**Table 3:** Vitamin D compared to fecal calprotectin (FCP) and other biomarkers

	All patient episodes (*n* = 325)	CD episodes (*n* = 184)	UC episodes (*n* = 127)
Vitamin D *vs* FCP	*r* = −0.19 (−0.29 to −0.080), *P* < 0.001	*r* = −0.19 (−0.33 to −0.046), *P* = 0.008	*r* = −0.23 (−0.39 to −0.052), *P* = 0.010
Vitamin D *vs* CRP	*r* = −0.16 (−0.27 to −0.05), *P* = 0.005	*r* = −0.13 (−0.27 to 0.03), *P* = 0.098	*r* = −0.16 (−0.34 to 0.024), *P* = 0.080
Vitamin D *vs* albumin	*r* = 0.080 (−0.035 to 0.19), *P* = 0.16	*r* = −0.010 (−0.16 to 0.14), *P* = 0.90	*r* = 0.22 (0.036 to 0.39), *P* = 0.016
Vitamin D *vs* platelets	*r* = − 0.15 (−0.26 to −0.039), *P* = 0.007	*r* = −0.14 (−0.28 to 0.016), *P* = 0.072	*r* = − 0.21 (−0.38 to −0.033), *P* = 0.017
Vitamin D *vs* hemoglobin	*r* = 0.048 (−0.066 to 0.16), *P* = 0.40	*r* = −0.041 (−0.19 to 0.11), *P* = 0.58	*r* = 0.17 (−0.016 to 0.34), *P* = 0.066
Vitamin D *vs* CRP/albumin ratio	*r* = −0.150 (−0.26 to −0.031), *P* = 0.011	*r* = −0.10 (−0.25 to 0.057), *P* = 0.20	*r* = −0.22 (−0.39 to −0.029), *P* = 0.020

CD, Crohn's disease; CRP, C‐reactive protein; UC, ulcerative colitis.

Vitamin D levels were also compared against clinical scores for CD (the HBI) and UC (the pMayo score). Vitamin D level in CD did not significantly correlate with HBI (*r* = −0.29 [−0.18 to 0.12], *P* = 0.70) but it did correlate with pMayo in the UC cohort (*r* = −0.24 [−0.40 to −0.060], *P* = 0.007).

An analysis of vitamin D level compared to whether FCP was elevated or normal (with elevated set at 150 μg/g) was carried out. One‐hundred and fifty‐three patient episodes had FCP >150 μg/g. Area under the ROC curve was 0.58.

**Figure 2:**
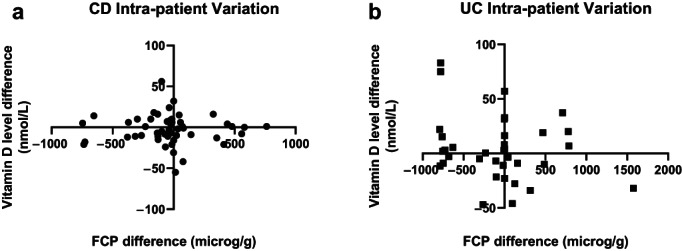
Intra‐patient variation for Crohn's disease (CD—a) and ulcerative colitis (UC—b) subsets.

The ratio of vitamin D level to FCP level was calculated and compared for each CD and UC patient episode with two different time points, at any stage during the study period. Fifty patients with CD had more than one time point of matched data. When change in FCP was compared with change in vitamin D level using Spearman's correlation, no significant difference was seen (*r* = −0.056, 95% CI: –0.34 to 0.23). Twenty‐six of 50 patients (52%) had concordant changes (FCP decreased with increasing vitamin D, or vice versa). Thirty‐five patients with UC had more than one time point of matched data. When change in FCP was compared with change in vitamin D level using Spearman's correlation, no significant difference was seen (*r* = −0.14, 95% CI: –0.46 to 0.21). Eighteen of the 35 patients (51%) had concordant changes in their vitamin D levels compared to their FCP (Fig. 2). Wilcoxon matched‐pair signed‐rank test revealed that there was no significant difference within individual patients when comparing their ratio and change over two time points in both the CD and UC disease subsets (*P* = 0.16 for both groups).

## Discussion

This real‐world retrospective study assessing the potential role of vitamin D as a biomarker in IBD showed a significant inverse correlation between vitamin D and FCP levels in both CD and UC. This adds further support for the existing evidence of vitamin D as a negative acute‐phase reactant.^14^, ^22^ Despite the correlation between low vitamin D and elevated FCP in this cohort, no clinically significant cut‐off vitamin D level to indicate active inflammation could be determined from the ROC curve analysis. This observation did not consider the season of testing or patients taking vitamin D supplementation.

When vitamin D was considered against other biomarkers, correlation was predominantly seen in the disease subgroups. Vitamin D level correlated with albumin in UC but not in CD. CRP and CRP/albumin ratio also correlated with vitamin D levels in UC patients. Low vitamin D has been shown to correlate with reduced serum albumin, particularly in renal disease and albuminuria,^23^ but no cause for the correlations in UC more so than CD is immediately evident, particularly as more patient episodes were CD. Interestingly, CRP is not always thought to correspond to UC activity as accurately as CD,^1^, ^24^ but the correlation between vitamin D level and CRP was not statistically significant in CD but it was in UC. Vitamin D levels in CD did not significantly correlate with HBI. This is in contrast to previously published data demonstrating low vitamin D levels in those with elevated HBI.^25^

No significant intra‐patient change in vitamin D level was noted in the 85 patients with multiple matched data points. Intra‐patient variability was difficult to assess from this retrospective chart analysis. The majority of patients with multiple data points had stable medication throughout the study period. Patients with two time points of data were assessed with the difference between their vitamin D and calprotectin levels. Although these differences were not significant, this is the first study to examine these datasets in both CD and UC. Both sets of data showed concordance of data (FCP decreased with increasing vitamin D, or vice versa) in about 50% of patients. This was the more expected outcome given vitamin D appears to be a negative acute‐phase reactant. Reasons as to why it did not occur in the other half of patients may include vitamin D supplementation or appropriate exposure, as vitamin D deficiency is a known problem in Australia and is routinely mentioned and measured in this IBD clinic.

Low vitamin D is common, particularly in temperate climates. In Australia, vitamin D deficiency has been described as severe with a level of <13 nmol/L, moderate with a level of 13–29 nmol/L, and mild with a level of 30–49 nmol/L. A level of ≥50 nmol/L is considered adequate.^26^ In contrast to European data showing vitamin D insufficiency or deficiency in 80% of IBD patients at diagnosis,^25^ median vitamin D levels in our cohort during clinical monitoring were adequate when considered against Australian recommendations.^26^ Conversely, data from slightly further south in Australia (Tasmania) in an autoimmune disease cohort (multiple sclerosis) and control populations showed a high prevalence of vitamin D deficiency.^27^ This further supports routine monitoring of vitamin D in patients with IBD living in a temperate climate.

The strength of this study was the large, well‐characterized representative cohort with multiple time points for analysis. There were more patients on biologic therapy in our cohort compared to previous international cohorts examining disease activity,^28^ and many patients had active disease episodes. All patients lived in a temperate‐climate city with limited sun exposure over winter.

Several limitations existed in this study. First, this was a retrospective, single‐center study using FCP as a surrogate gold standard for disease activity. Endoscopic results were not recorded because of the noninvasive and clinic‐based nature of the study. Data on vitamin D supplementation were not recorded for all patients, particularly as it is commonly an over‐the‐counter medication. Adherence to prescribed vitamin D supplements was also not uniformly assessed. Although smoking has been noted to be associated with lower vitamin D levels in CD patients,^12^ the overall number of smokers was small and not all patients had their smoking status recorded. Additionally, smoking can impact vitamin D status, leading to lower vitamin D levels.^25^, ^29^ The effect of this was not possible to account for, given the paucity of reliable smoking data. Ethnicity and skin type were not recorded from the chart review but would play a role in individuals' ability to absorb environmental vitamin D. A future prospective study would allow for these missing data to be collected; biologic response and vitamin D levels may also be compared. The choice of the 3‐month timeframe for a matched dataset of bloods and calprotectin allowed a greater number of episodes to be analyzed, but it may have reduced the correlation between FCP and serum biomarker results. Finally, the main laboratory processing the FCP had an upper limit of detection at 800 μg/g. Many of the more elevated results were limited by this restriction. This limitation of the upper limit was removed in the laboratory in 2020, which will improve the accuracy of future studies.

## Conclusion

Overall, this study shows support for an inverse correlation between vitamin D levels and FCP levels in IBD patients and in both the CD and UC subsets. Vitamin D did not meet criteria for a sensitive and specific biomarker. Vitamin D levels had more variable correlation with serum biomarkers. There was no significant correlation with any of the other biomarkers tested in CD; however, in UC a statistically significant correlation did exist for vitamin D level and serum albumin, platelets, and CRP/albumin ratio. Overall, vitamin D may be a useful addition to existing biomarkers, particularly in UC.

Prospective evaluation of vitamin D levels with concurrent blood and FCP in a larger cohort is required to ascertain when changes in vitamin D occur. Larger prospective studies may confer more robust results. Further studies assessing the incidence of new vitamin D deficiency in patients with active inflammation compared to patients in remission and the response to replacement in patients with IBD flares compared to non‐inflamed patients would be of benefit.

## Ethics statement

This study protocol (RES‐19‐0000‐957Q) was reviewed and approved by Monash Health Human Research and Ethics Committee, an institutional ethics committee, as part of quality and service improvement. Explicit patient consent was not required, as clinically necessary data were collected retrospectively and de‐identified.

## Data Availability

The data that support the findings of this study are not publicly available due to patient confidentiality, but are available from the corresponding author upon reasonable request.

## References

[1] Turner D , Ricciuto A , Lewis A *et al*. International Organization for the STRIDE‐II: An Update on the Selecting Therapeutic Targets in Inflammatory Bowel Disease (STRIDE) Initiative of the International Organization for the Study of IBD (IOIBD): Determining Therapeutic Goals for Treat‐to‐Target strategies in IBD. Gastroenterology. 2021; 160: 1570–1583. 33359090 10.1053/j.gastro.2020.12.031

[2] Vermeire S , Van Assche G , Rutgeerts P . C‐reactive protein as a marker for inflammatory bowel disease. Inflamm. Bowel Dis. 2004; 10: 661–665. 15472532 10.1097/00054725-200409000-00026

[3] Gibson DJ , Hartery K , Doherty J *et al*. CRP/albumin ratio: an early predictor of steroid responsiveness in acute severe ulcerative colitis. J. Clin. Gastroenterol. 2018; 52: e48–e52. 28737646 10.1097/MCG.0000000000000884

[4] Mosli MH , Zou G , Garg SK *et al*. C‐reactive protein, fecal calprotectin, and stool lactoferrin for detection of endoscopic activity in symptomatic inflammatory bowel disease patients: a systematic review and meta‐analysis. Am. J. Gastroenterol. 2015; 110: 802–819. 25964225 10.1038/ajg.2015.120

[5] Kalla R , Boyapati R , Vatn S *et al*. Patients' perceptions of fecal calprotectin testing in inflammatory bowel disease: results from a prospective multicenter patient‐based survey. Scand. J. Gastroenterol. 2018; 53: 1437–1442. 30451040 10.1080/00365521.2018.1527394

[6] Maaser C , Sturm A , Vavricka SR *et al*. ECCO‐ESGAR Guideline for Diagnostic Assessment in IBD Part 1: initial diagnosis, monitoring of known IBD, detection of complications. J. Crohns Colitis. 2019; 13: 144–164. 30137275 10.1093/ecco-jcc/jjy113

[7] Asthana AK , Friedman AB , Maconi G *et al*. The failure of gastroenterologists to apply intestinal ultrasound in inflammatory bowel disease in the Asia‐Pacific: a need for action. J. Gastroenterol. Hepatol. (Australia). 2015; 30: 446–452. 10.1111/jgh.12871 25529767

[8] Ardesia M , Ferlazzo G , Fries W , Šebeková K . Vitamin D and inflammatory bowel disease. Biomed. Res. Int. 2015; 2015: 1–16. 10.1155/2015/470805 PMC4427008 26000293

[9] Xiao D , Zhang X , Ying J *et al*. Association between vitamin D status and sepsis in children: a meta‐analysis of observational studies. Clin. Nutr. 2019; 39: 1735–1741. 31495735 10.1016/j.clnu.2019.08.010

[10] Yin K , Agrawal D . Vitamin D and inflammatory diseases. J. Inflamm. Res. 2014; 7: 69–87. 24971027 10.2147/JIR.S63898 PMC4070857

[11] Garg M , Royce SG , Tikellis C *et al*. The intestinal vitamin D receptor in inflammatory bowel disease: inverse correlation with inflammation but no relationship with circulating vitamin D status. Therap. Adv. Gastroenterol. 2019; 12: 1756284818822566. 10.1177/1756284818822566 PMC6348511 30719077

[12] Jørgensen SP , Hvas CL , Agnholt J , Christensen LA , Heickendorff L , Dahlerup JF . Active Crohn's disease is associated with low vitamin D levels. J. Crohns Colitis. 2013; 7: e407–e413. 23403039 10.1016/j.crohns.2013.01.012

[13] Ghaly S , Murray K , Baird A *et al*. High vitamin D‐binding protein concentration, low albumin, and mode of remission predict relapse in Crohn's disease. Inflamm. Bowel Dis. 2016; 22: 2456–2464. 27631600 10.1097/MIB.0000000000000894

[14] Haifer C , Lawrance IC , Center JR *et al*. Vitamin D metabolites are lower with active Crohn's disease and spontaneously recover with development of remission. Therap. Adv. Gastroenterol. 2019; 12: 1756284819865144. 10.1177/1756284819865144 PMC6661794 31384306

[15] Garg M , Rosella O , Lubel J , Gibson P . P331 Total, free and bioavailable 25(OH) vitamin D inversely correlate with fecal calprotectin in patients with inflammatory bowel disease. J. Crohns Colitis. 2013; 7: S142.

[16] Fletcher J , Cooper SC , Ghosh S , Hewison M . The role of vitamin D in inflammatory bowel disease: mechanism to management. Nutrients. 2019; 11: 1019. 31067701 10.3390/nu11051019 PMC6566188

[17] López‐Muñoz P , Beltrán B , Sáez‐González E , Alba A , Nos P , Iborra M . Influence of vitamin D deficiency on inflammatory markers and clinical disease activity in IBD patients. Nutrients. 2019; 11: 1059. 31083541 10.3390/nu11051059 PMC6567866

[18] Nicholson I , Dalzell AM , El‐matary W . Vitamin D as a therapy for colitis: a systematic review. J. Crohns Colitis. 2012; 6: 405–411. 22398085 10.1016/j.crohns.2012.01.007

[19] Li J , Chen N , Wang D , Zhang J , Gong X . Efficacy of vitamin D in treatment of inflammatory bowel disease: a meta‐analysis. Medicine. 2018; 97: e12662. 30431562 10.1097/MD.0000000000012662 PMC6257592

[20] Chemical Pathology Advisory Committee . Position Statement. Royal College of Pathologists of Australasia, 2019; 1–8.

[21] Sandborn WJ , Panés J , Zhang H , Yu D , Niezychowski W , Su C . Correlation between concentrations of fecal calprotectin and outcomes of patients with ulcerative colitis in a phase 2 trial. Gastroenterology. 2016; 150: 96–102. 26376350 10.1053/j.gastro.2015.09.001

[22] Meckel K , Li YC , Lim J *et al*. Serum 25‐hydroxyvitamin D concentration is inversely associated with mucosal inflammation in patients with ulcerative colitis. Am. J. Clin. Nutr. 2016; 104: 113–120. 27281309 10.3945/ajcn.115.123786 PMC4919525

[23] Isakova T , Gutiérrez OM , Patel NM , Andress DL , Wolf M , Levin A . Vitamin D deficiency, inflammation, and albuminuria in chronic kidney disease: complex interactions. J. Ren. Nutr. 2011; 21: 295–302. 20817560 10.1053/j.jrn.2010.07.002

[24] Cioffi M . Laboratory markers in ulcerative colitis: Current insights and future advances. World J. Gastrointest. Pathophysiol. 2015; 6: 13–22. 25685607 10.4291/wjgp.v6.i1.13 PMC4325297

[25] Chetcuti Zammit S , Ellul P , Girardin G *et al*. Vitamin D deficiency in a European inflammatory bowel disease inception cohort: an Epi‐IBD study. Eur. J. Gastroenterol. Hepatol. 2018; 30: 1297–1303. 30134383 10.1097/MEG.0000000000001238

[26] Australian Bureau of Statistics . Vitamin D. Canberra: Australian Bureau of Statistics, 2013.

[27] van der Mei IAF , Ponsonby AL , Dwyer T *et al*. Vitamin D levels in people with multiple sclerosis and community controls in Tasmania, Australia. J. Neurol. 2007; 254: 581–590. 17426912 10.1007/s00415-006-0315-8

[28] Mak WY , Zhao M , Ng SC , Burisch J . The epidemiology of inflammatory bowel disease: East meets west. J. Gastroenterol. Hepatol. (Australia). 2020; 35: 380–389. 10.1111/jgh.14872 31596960

[29] Brot C , Jùrgensen NR , Sùrensen OH . The influence of smoking on vitamin D status and calcium metabolism. European Journal of Clinical Nutrition. 1999; 53: 920–926. 10602348 10.1038/sj.ejcn.1600870

